# Ring Laser Gyro G-Sensitive Misalignment Calibration in Linear Vibration Environments

**DOI:** 10.3390/s18020601

**Published:** 2018-02-16

**Authors:** Lin Wang, Wenqi Wu, Geng Li, Xianfei Pan, Ruihang Yu

**Affiliations:** National University of Defense Technology, Changsha 410073, China; wanglin11@nudt.edu.cn (L.W.); lg_nudt@nudt.edu.cn (G.L.); panxianfei@nudt.edu.cn (X.P.); yuruihang@nudt.edu.cn (R.Y.)

**Keywords:** RLG, g-sensitive misalignments, linear vibration

## Abstract

The ring laser gyro (RLG) dither axis will bend and exhibit errors due to the specific forces acting on the instrument, which are known as g-sensitive misalignments of the gyros. The g-sensitive misalignments of the RLG triad will cause severe attitude error in vibration or maneuver environments where large-amplitude specific forces and angular rates coexist. However, g-sensitive misalignments are usually ignored when calibrating the strapdown inertial navigation system (SINS). This paper proposes a novel method to calibrate the g-sensitive misalignments of an RLG triad in linear vibration environments. With the SINS is attached to a linear vibration bench through outer rubber dampers, rocking of the SINS can occur when the linear vibration is performed on the SINS. Therefore, linear vibration environments can be created to simulate the harsh environment during aircraft flight. By analyzing the mathematical model of g-sensitive misalignments, the relationship between attitude errors and specific forces as well as angular rates is established, whereby a calibration scheme with approximately optimal observations is designed. Vibration experiments are conducted to calibrate g-sensitive misalignments of the RLG triad. Vibration tests also show that SINS velocity error decreases significantly after g-sensitive misalignments compensation.

## 1. Introduction

Strapdown inertial navigation systems (SINS) has been widely used in many applications. In order to improve the SINS accuracy, many techniques have been adopted, such as the zero velocity updated (ZUPT) technique [1], the data processing technique [2], and the Kalman filter technique [3,4]. A SINS based on Micro-Electro-Mechanical Syetem (MEMS) has also been used in some highly dynamic environments [5]. In high accuracy applications, the ring laser gyro (RLG) has become a common instrument in SINS for spacecraft and other aerospace systems [6]. In these applications, SINS may be employed in harsh working environments, e.g., vibration environments, maneuver motions with large-amplitude specific forces and angular rates, or other highly dynamic environments. The laser block of an RLG is mounted on the inertial measurement unit (IMU) base through a dither suspension structure. However, because the transverse stiffness of the RLG dither axis is limited [7], the RLG dither axis will bend and exhibit errors due to the specific forces acting on the instrument, which are known as g-sensitive misalignments of the gyros. Namely, the laser block of the RLG will tilt under the influence of specific forces. The g-sensitive error is usually taken into consideration when calibrating MEMS gyros [8,9]. However, the calibration of g-sensitive misalignments regarding high-accuracy RLGs is usually ignored for general applications [10,11]. In aerospace applications, due to the presence of great acceleration and angular rate, g-sensitive misalignments of the RLG triad will introduce equivalent gyro drift, causing further attitude error and velocity error. Hence, g-sensitive misalignments compensation must be taken into consideration in the SINS navigation software.

In order to reduce the influence of g-sensitive misalignments in a vibration environment, parameter optimization has been applied to the RLG dither suspension structure [12], and the vibration isolation structure of the SINS has been elaborately designed [13]. However, the vibration tests performed on the laser SINS for a Cyclone-4 launch vehicle still reveal that the performance degrades regardless of careful vibration isolation design, and equivalent gyro drift occurs in the SINS [14]. In References [15,16], a Kalman filter and a multi-position calibration method are adopted to estimate the RLG g-dependent biases; however, the method to calibrate g-sensitive misalignments of the RLG triad is not discussed. The influence of g-sensitive misalignment errors upon the estimation accuracy of SINS error parameters is verified via numerical simulations in Reference [17]. The drift errors caused by RLG dither axis bending are analyzed in Reference [18]; however, only theoretical and simulation results are discussed. The calibration and compensation methods are not given in Reference [18]. A calibration method is proposed in Reference [19], in which a sequence of coning tests on a high-precision three-axis turntable are conducted to estimate the g-sensitive misalignments of the RLGs caused by gravity; however, the procedures are very complicated and time-consuming. The calibration method proposed in Reference [18] is based on static tests and is only suitable in low-g (less than 1 g) environments. In addition, actual calibration experiments and vibration tests are not conducted.

This paper presents a novel method to calibrate the g-sensitive misalignments of an RLG triad in linear vibration environments. The g-sensitive misalignments of the RLG triad is deduced and the mechanism is analyzed. An equivalent gyro drift model is established to express the error caused by g-sensitive misalignments, which builds a relationship between the equivalent gyro drift and the specific forces as well as the angular rate. Further, the attitude error caused by g-sensitive misalignments is analyzed, whereby a calibration method based on vibration experiments is designed. To fully excite the errors caused by g-sensitive misalignments, vibration experiments are elaborately designed. With the SINS attached to a linear vibration bench through outer rubber dampers, rocking of the SINS can occur when the linear vibration bench is employed. Therefore, linear vibration environments can be created to simulate the harsh environment during aircraft flight. In this condition, g-sensitive misalignments of the RLG triad will cause severe attitude errors. By analyzing the mathematical model of g-sensitive misalignments of an RLG triad, the attitude error observation equations are deduced. With error parameter sensitivity maximized, a calibration scheme with approximately optimal observations is designed. Experiments are also conducted to validate the method proposed in the paper.

## 2. Mathematical and Model 

**Figure 1:**
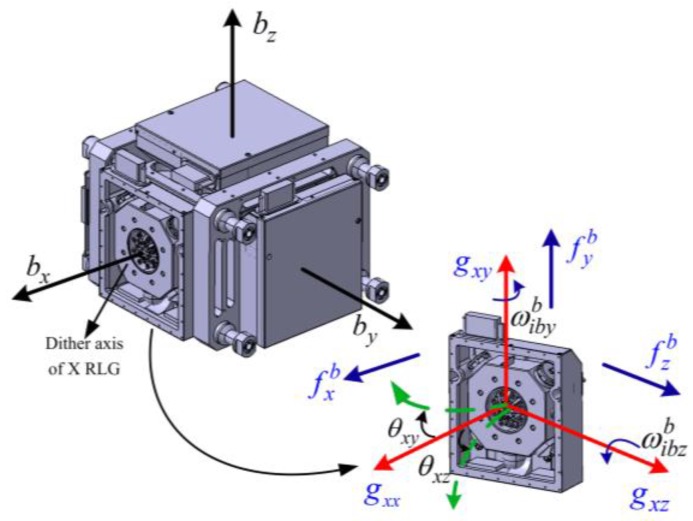
Bending of the ring laser gyro (RLG) dither axis and corresponding g-sensitive misalignments.

Because of the dither suspension structure, the RLG dither axis will bend and exhibit errors due to the specific forces acting on the instrument, which are known as g-sensitive misalignments of the gyros. The bending of *X* RLG dither axis under specific forces, depicted in Figure 1, is taken as an example to explain the mechanism of g-sensitive misalignments. As shown in Figure 1, the *X* RLG body coordinate frame-$b_{g_{x}}$ is defined as follows: $g_{xx}$-axis coincides with the *X* gyro sensitive axis, namely, the $b_{x}$-axis of the SINS body frame-b; $g_{xy}$ and $g_{xz}$-axes are parallel to the $b_{y}$ and $b_{z}$-axes of the SINS body frame, respectively. The *X* RLG body coordinate frame-$b_{g_{x}}$ and SINS body frame-b are right-hand orthogonal coordinate frames. They are nominally parallel. 

In general, the frame-$b_{g_{x}}$ is parallel to the SINS body frame-*b*. However, if the specific forces act on the *X* RLG, the aforementioned two coordinate frames will no longer be parallel. There will be a small angle between them. A rotation vector denoted by $\theta_{g_{x}}$ can be used to express the rotation from the frame-*b* to the frame-$b_{g_{x}}$, and is given by:

(1) $$\theta_{g_{x}}={\left[\begin{array}{ccc} 0 & -\theta_{xz} & \theta_{xy} \end{array}\right]}^{T}$$

where $\theta_{xz}$ and $\theta_{xy}$ respectively denote the *X* gyro-sensitive axis misalignment toward $b_{z}$ and $b_{y}$-axes of the SINS due to specific forces acting on the *X* RLG. The quantity of $\theta_{g_{x}}$ is a small angle. The angular rate of *X* RLG expressed in the frame-$b_{g_{x}}$ is given by:

(2) $$\omega_{ib_{g_{x}}}^{b_{g_{x}}}=C_{b}^{b_{g_{x}}}\omega_{ib}^{b}$$

where $\omega_{ib_{g_{x}}}^{b_{g_{x}}}$ is the angular rate of *X* RLG expressed in the frame-$b_{g_{x}}$, $\omega_{ib}^{b}$ is the true angular rate of the SINS expressed in the frame-b, and $C_{b}^{b_{g_{x}}}$ is the direction cosine matrix from the frame-b to the frame-$b_{g_{x}}$.

Based on small angle assumption, the direction cosine matrix from the frame-b to the frame-$b_{g_{x}}$, i.e., $C_{b}^{b_{g_{x}}}$, can be expressed as $C_{b}^{b_{g_{x}}}\approx I_{3}-\theta_{g_{x}}\times$. Hence, Equation (2) can be rewritten as:

(3) $$\begin{array}{cc} \omega_{ib_{g_{x}}}^{b_{g_{x}}} & =\left(I_{3}-\theta_{g_{x}}\times \right)\omega_{ib}^{b}\\ & =\omega_{ib}^{b}-\theta_{g_{x}}\times \omega_{ib}^{b} \end{array}$$

where $I_{3}$ denotes a unit matrix, and $\theta_{g_{x}}\times$ denotes the skew symmetric matrix of vector $\theta_{g_{x}}$.

Rewriting Equation (3) in components form leads to another identical expression:

(4) $$\omega_{ib_{g_{x}}}^{b_{g_{x}}}=\left[\begin{array}{c} \omega_{ibx}^{b}+\omega_{iby}^{b}\theta_{xy}+\omega_{ibz}^{b}\theta_{xz}\\ \omega_{iby}^{b}-\omega_{ibx}^{b}\theta_{xy}\\ \omega_{ibz}^{b}-\omega_{ibx}^{b}\theta_{xz} \end{array}\right]$$

In fact, only the first component of Equation (4) can be sensed by *X* RLG. The term $\omega_{iby}^{b}\theta_{xy}+\omega_{ibz}^{b}\theta_{xz}$ denotes the equivalent gyro drift caused by the g-sensitive misalignments of *X* RLG. To clarify, we use ${\tilde{\omega }}_{ibx}^{b}$ to represent the angular rate sensed by *X* RLG, i.e., the first component of $\omega_{ib_{g_{x}}}^{b_{g_{x}}}$. Hence, we obtain:

(5) $${\tilde{\omega }}_{ibx}^{b}=\omega_{ibx}^{b}+\omega_{iby}^{b}\theta_{xy}+\omega_{ibz}^{b}\theta_{xz}$$

Analogously, the angular rates sensed by *Y* RLG and *Z* RLG are respectively given by:

(6) $${\tilde{\omega }}_{iby}^{b}=\omega_{iby}^{b}+\omega_{ibx}^{b}\theta_{yx}+\omega_{ibz}^{b}\theta_{yz}$$

(7) $${\tilde{\omega }}_{ibz}^{b}=\omega_{ibz}^{b}+\omega_{ibx}^{b}\theta_{zx}+\omega_{iby}^{b}\theta_{zy}$$

where ${\tilde{\omega }}_{iby}^{b}$ and ${\tilde{\omega }}_{ibz}^{b}$ respectively denote the angular rate sensed by *Y* RLG and *Z* RLG, and $\theta_{ij}$ (*i* = *x*, *y*, *z*; *j* = *x*, *y*, *z*; *i* ≠ *j*) denotes the *i* gyro misalignment toward the *j*-axis due to specific forces acting on the *i* gyro. 

According to Equations (5)–(7), the mathematical model for the g-sensitive misalignments of an RLG triad can be represented as:

(8) $$\delta \omega_{ib}^{b}={\tilde{\omega }}_{ib}^{b}-\omega_{ib}^{b}=M\omega_{ib}^{b}$$

with

(9) $${\tilde{\omega }}_{ib}^{b}=\left[\begin{array}{c} {\tilde{\omega }}_{ibx}^{b}\\ {\tilde{\omega }}_{iby}^{b}\\ {\tilde{\omega }}_{ibz}^{b} \end{array}\right],M=\left[\begin{array}{ccc} 0 & \theta_{xy} & \theta_{xz}\\ \theta_{yx} & 0 & \theta_{yz}\\ \theta_{zx} & \theta_{zy} & 0 \end{array}\right]$$

where $\delta \omega_{ib}^{b}$ denotes the equivalent gyro drift of the RLG triad expressed in the body frame-*b*, ${\tilde{\omega }}_{ib}^{b}$ denotes the angular rate sensed by the RLG triad, and M denotes the g-sensitive misalignments matrix of the RLG triad. 

As shown in References [18,19], the g-sensitive misalignments in Equation (9) are proportional to the amount of specific force acting on the RLG, which can be expressed as:

(10) $$\left[\begin{array}{c} \theta_{xy}\\ \theta_{xz} \end{array}\right]=\left[\begin{array}{c} f_{x}^{b}\tau_{xyx}+f_{y}^{b}\tau_{xyy}\\ f_{x}^{b}\tau_{xzx}+f_{z}^{b}\tau_{xzz} \end{array}\right],\left[\begin{array}{c} \theta_{yx}\\ \theta_{yz} \end{array}\right]=\left[\begin{array}{c} f_{x}^{b}\tau_{yxx}+f_{y}^{b}\tau_{yxy}\\ f_{y}^{b}\tau_{yzy}+f_{z}^{b}\tau_{yzz} \end{array}\right],\left[\begin{array}{c} \theta_{zx}\\ \theta_{zy} \end{array}\right]=\left[\begin{array}{c} f_{x}^{b}\tau_{zxx}+f_{z}^{b}\tau_{zxz}\\ f_{y}^{b}\tau_{zyy}+f_{z}^{b}\tau_{zyz} \end{array}\right]$$

where $f_{i}^{b}$ (*i* = *x*, *y*, *z*) denotes the *i*-axis specific force sensed by accelerometer triad, and $\tau_{ijk}$ (*i* = *x*, *y*, *z*; *j* = *x*, *y*, *z*; *k* = *x*, *y*, *z*; *i* ≠ *j*) denotes the flexure coefficient which accounts for *i* gyro misalignment toward the *j*-axis due to specific force along *k*-axis, whose unit is rad/(m/s^2^) or arc-second/g (1 g ≈ 9.8 m/s^2^). 

Substituting Equations (9) and (10) into Equation (8) leads to:

(11) $$\delta \omega_{ib}^{b}=\left[\begin{array}{ccc} 0 & f_{x}^{b}\tau_{xyx}+f_{y}^{b}\tau_{xyy} & f_{x}^{b}\tau_{xzx}+f_{z}^{b}\tau_{xzz}\\ f_{x}^{b}\tau_{yxx}+f_{y}^{b}\tau_{yxy} & 0 & f_{y}^{b}\tau_{yzy}+f_{z}^{b}\tau_{yzz}\\ f_{x}^{b}\tau_{zxx}+f_{z}^{b}\tau_{zxz} & f_{y}^{b}\tau_{zyy}+f_{z}^{b}\tau_{zyz} & 0 \end{array}\right]\omega_{ib}^{b}$$

where $\omega_{ib}^{b}$ can be expressed in components form as $\omega_{ib}^{b}={\left[\begin{array}{ccc} \omega_{ibx}^{b} & \omega_{iby}^{b} & \omega_{ibz}^{b} \end{array}\right]}^{T}$.

Equation (11) is the equivalent gyro drift induced by the g-sensitive misalignments of an RLG triad. Twelve flexure coefficients notated as a vector τ are contained in Equation (11), and τ is given by:

(12) $$\tau ={\left[\begin{array}{cccccccccccc} \tau_{xyx} & \tau_{xyy} & \tau_{xzx} & \tau_{xzz} & \tau_{yxx} & \tau_{yxy} & \tau_{yzy} & \tau_{yzz} & \tau_{zxx} & \tau_{zxz} & \tau_{zyy} & \tau_{zyz} \end{array}\right]}^{T}$$

The task of this paper is to estimate these parameters and compensate the corresponding errors. Substituting Equation (12) into Equation (11) and rearranging Equation (11) leads to:

(13) $$\delta \omega_{ib}^{b}\approx \Gamma (t)\tau$$

with

(14) $$\begin{array}{l} \Gamma (t)=\left[\begin{array}{ccc} \Gamma_{1} & 0_{1\times 4} & 0_{1\times 4}\\ 0_{1\times 4} & \Gamma_{2} & 0_{1\times 4}\\ 0_{1\times 4} & 0_{1\times 4} & \Gamma_{3} \end{array}\right]\\ \Gamma_{1}=\left[\begin{array}{cccc} \omega_{iby}^{b}f_{x}^{b} & \omega_{iby}^{b}f_{y}^{b} & \omega_{ibz}^{b}f_{x}^{b} & \omega_{ibz}^{b}f_{z}^{b} \end{array}\right],\Gamma_{2}=\left[\begin{array}{cccc} \omega_{ibx}^{b}f_{x}^{b} & \omega_{ibx}^{b}f_{y}^{b} & \omega_{ibz}^{b}f_{y}^{b} & \omega_{ibz}^{b}f_{z}^{b} \end{array}\right]\\ \Gamma_{3}=\left[\begin{array}{cccc} \omega_{ibx}^{b}f_{x}^{b} & \omega_{ibx}^{b}f_{z}^{b} & \omega_{iby}^{b}f_{y}^{b} & \omega_{iby}^{b}f_{z}^{b} \end{array}\right] \end{array}$$

## 3. Calibration Method

In this section, a calibration algorithm based on vibration tests to estimate unknown parameters in Equation (13) is introduced, followed by a calibration scheme designed with approximately optimal observations.

### 3.1. Calibration Algorithm

Based on the attitude update algorithm, the direction cosine matrix expressing the rotation from the body frame-*b* to the inertial frame-*i* can be updated by the gyro output $\omega_{ib}^{b}$. The attitude update algorithm is given by:

(15) $${\dot{C}}_{b}^{i}=C_{b}^{i}[\omega_{ib}^{b}\times ],C_{b}^{i}\left(t_{0}\right)≜C_{b\left(t_{0}\right)}^{b\left(t_{0}\right)}=I_{3}$$

where $t_{0}$ is the start time of vibration, and the inertial frame-*i* is formed by fixing the *b*-frame at the time $t_{0}$ in the inertial space, namely, $i≜b(t_{0})$.

If there is no vibration acting on the SINS, the calculated attitude according to Equation (15) is without error. However, due to the linear vibration acting on the SINS, g-sensitive misalignments will occur. As a result, the attitude calculated by Equation (15) will exhibit great error introduced by equivalent gyro drift. The time derivative of attitude error is given by:

(16) $${\dot{\phi }}^{i}=-C_{b}^{i}\delta \omega_{ib}^{b}$$

where $\phi^{i}$ denotes the attitude error expressed in the inertial frame-*i*.

Substituting Equation (13) into Equation (16), taking the integral of Equation (16) from time $t_{0}$ to time $t_{k}$, and rearranging it leads to:

(17) $$\phi^{i}(t_{k})=\left[-\int_{t_{0}}^{t_{k}}C_{b}^{i}(t)\Gamma (t)dt\right]\tau$$

where $t_{k}$ is the end time of the vibration. Here, the initial attitude error in Equation (17) is ignorable, namely, $\phi^{i}(t_{0})\approx 0_{3\times 1}$. According to Equation (17), τ can be estimated with attitude error observations. Calibration experiments can be conducted through following steps:

- Step 1: The SINS is first at rest for a certain time to carry out static self-alignment. The static self-alignment phase provides the initial attitude, which is essential information to make the SINS work. The initial attitude is not influenced by g-sensitive misalignments.
- Step 2: The linear vibration is applied to the SINS following the static period, and lasts a few minutes, e.g., 8–10 min. The SINS works on inertial navigation mode. Based on the initial attitude calculated by Step 1, the SINS attitude is updated to the end of vibration. During this period, by an elaborately designed fixed device, the attitude error of SINS is stimulated under the influence of g-sensitive misalignments.
- Step 3: The SINS returns to the stationary state and performs static self-alignment again. This phase is only employed to provide the correct attitude reference of SINS. Based on the attitude reference calculated in Step 3, the error of attitude calculated by Step 2 can be observed and is given by:
  (18) $$\left[\phi_{O}^{i}(t_{k})\times \right]=I_{3}-{\tilde{C}}_{b}^{i}(t_{k})C_{i}^{b}(t_{k})$$
  with
  (19) $$C_{i}^{b}(t_{k})≜C_{b(t_{0})}^{b(t_{k})}=C_{n(t_{k})}^{b(t_{k})}C_{n(t_{0})}^{n(t_{k})}C_{b(t_{0})}^{n(t_{0})}$$
  (20) $$\begin{array}{cc} C_{n(t_{0})}^{n(t_{k})} & =I_{3}-\frac{\sin[\omega_{ie}(t_{k}-t_{0})]}{\omega_{ie}(t_{k}-t_{0})}\left[(\omega_{ie}^{n}(t_{k}-t_{0}))\times \right]+\frac{1-\cos[\omega_{ie}(t_{k}-t_{0})]}{{\left[\omega_{ie}(t_{k}-t_{0})\right]}^{2}}{\left[(\omega_{ie}^{n}(t_{k}-t_{0}))\times \right]}^{2}\\ & =\left[\begin{array}{ccc} \cos[\omega_{ie}(t_{k}-t_{0})] & \sin(L)\sin[\omega_{ie}(t_{k}-t_{0})] & -\cos(L)\sin[\omega_{ie}(t_{k}-t_{0})]\\ -\sin(L)\sin[\omega_{ie}(t_{k}-t_{0})] & \sin^{2}(L)\cos[\omega_{ie}(t_{k}-t_{0})]+cos^{2}(L) & \sin(L)\cos(L)[1-\cos[\omega_{ie}(t_{k}-t_{0})]]\\ \cos(L)\sin[\omega_{ie}(t_{k}-t_{0})] & \sin(L)\cos(L)[1-\cos[\omega_{ie}(t_{k}-t_{0})]] & \mathrm{co}s^{2}(L)\cos[\omega_{ie}(t_{k}-t_{0})]+\sin^{2}(L) \end{array}\right] \end{array}$$
  where $\phi_{O}^{i}(t_{k})$ denotes the attitude error observation at time $t_{k}$, ${\tilde{C}}_{b}^{i}(t_{k})$ is the direction cosine matrix calculated by inertial navigation, and $C_{i}^{b}(t_{k})$ denotes the true direction cosine matrix at time $t_{k}$ calculated by Equation (19). $C_{n(t_{k})}^{b(t_{k})}$ is calculated by the realignment process in Step 3, $C_{b(t_{0})}^{n(t_{0})}$ is calculated by the alignment process in Step 1, and $C_{n(t_{0})}^{n(t_{k})}$ is calculated by Equation (20) with $\omega_{ie}^{n}={\left[\begin{array}{ccc} 0 & \omega_{ie}\cos(L) & \omega_{ie}\sin(L) \end{array}\right]}^{T}$ being the Earth’s rotation rate, $\omega_{ie}$ the Earth rate, and L the geographic latitude. Note that $C_{n(t_{0})}^{n(t_{k})}$ is determined by Earth’s rotation rate and vibration time, and there exists an explicit relationship with them.

**Figure 2:**
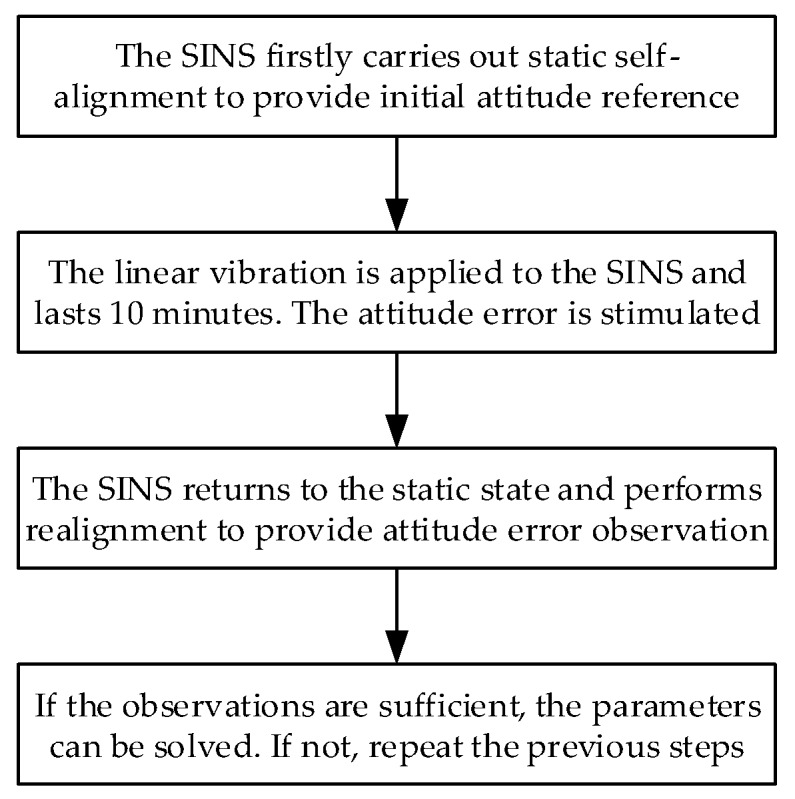
Calibration method process.

At least 12 different attitude error observations are required to solve the unknown parameters in Equation (17), so several vibration experiments are needed. Figure 2 shows the calibration method process. Considering the limitation of the north-finding accuracy, only the horizontal components of the attitude error observations in Equation (18) are used. Equation (17) can be solved by least squares as follows:

(21) $$\tau ={\left(A^{T}A\right)}^{-1}A^{T}B$$

with

(22) $$A=\left[\begin{array}{c} D{\left[-\int_{t_{0}}^{t_{k}}C_{b}^{i}(t)\Gamma (t)dt\right]}_{1}\\ D{\left[-\int_{t_{0}}^{t_{k}}C_{b}^{i}(t)\Gamma (t)dt\right]}_{2}\\ \vdots \\ D{\left[-\int_{t_{0}}^{t_{k}}C_{b}^{i}(t)\Gamma (t)dt\right]}_{n} \end{array}\right],B=\left[\begin{array}{c} D\phi_{O1}^{i}(t_{k})\\ D\phi_{O2}^{i}(t_{k})\\ \vdots \\ D\phi_{On}^{i}(t_{k}) \end{array}\right],D=\left[\begin{array}{ccc} 1 & 0 & 0\\ 0 & 1 & 0 \end{array}\right]$$

where the subscript 1, 2, …, *n* denotes the vibration experiment number. 

### 3.2. Optimal Observations

As mentioned in Reference [20], a good observation scheme not only allows all of the unknown parameters to be estimated, but also improves the accuracy of estimation. It is important to maximize the sensitivity of the attitude error observations with respect to these parameters.

Because the influence of the Earth’s rotation rate is ignorable in a short time, i.e., $C_{n(t)}^{n(t_{0})}\approx I_{3}$, and the attitude change induced by linear vibration satisfies small-angle approximation, i.e., $C_{b(t)}^{b(t_{0})}=C_{n(t_{0})}^{b(t_{0})}C_{n(t)}^{n(t_{0})}C_{b(t)}^{n(t)}\approx I_{3}$, Equation (17) can be simplified as:

(23) $$\phi^{i}(t_{k})==\left[-\int_{t_{0}}^{t_{k}}C_{b}^{i}(t)\Gamma (t)dt\right]\tau =\left[-\int_{t_{0}}^{t_{k}}C_{n(t_{0})}^{b(t_{0})}C_{n(t)}^{n(t_{0})}C_{b(t)}^{n(t)}\Gamma (t)dt\right]\tau \approx \left[-\int_{t_{0}}^{t_{k}}\Gamma (t)dt\right]\tau$$

Taking the partial derivatives of Equation (23) with respect to the components of τ leads to the Jacobian matrix, given as:

(24) $$\frac{\partial \phi^{i}(t_{k})}{\partial \tau }=-\int_{t_{0}}^{t_{k}}\Gamma (t)dt$$

We take *X* RLG as an example to illustrate how to obtain the optimal observations for its flexure coefficients. The first four columns of Equation (24) are the partial derivatives of $\phi^{i}(t_{k})$ with respect to $\tau_{xyx}$, $\tau_{xyy}$, $\tau_{xzx}$, and $\tau_{xzz}$.

(25) $$\frac{\partial \phi^{i}(t_{k})}{\partial \tau_{xyx}}={\left[\begin{array}{ccc} \int_{t_{0}}^{t_{k}}\omega_{iby}^{b}f_{x}^{b}dt & 0 & 0 \end{array}\right]}^{T},\frac{\partial \phi^{i}(t_{k})}{\partial \tau_{xzx}}={\left[\begin{array}{ccc} \int_{t_{0}}^{t_{k}}\omega_{ibz}^{b}f_{x}^{b}dt & 0 & 0 \end{array}\right]}^{T}$$

(26) $$\frac{\partial \phi^{i}(t_{k})}{\partial \tau_{xyy}}={\left[\begin{array}{ccc} \int_{t_{0}}^{t_{k}}\omega_{iby}^{b}f_{y}^{b}dt & 0 & 0 \end{array}\right]}^{T},\frac{\partial \phi^{i}(t_{k})}{\partial \tau_{xzz}}={\left[\begin{array}{ccc} \int_{t_{0}}^{t_{k}}\omega_{ibz}^{b}f_{z}^{b}dt & 0 & 0 \end{array}\right]}^{T}$$

**Figure 3:**
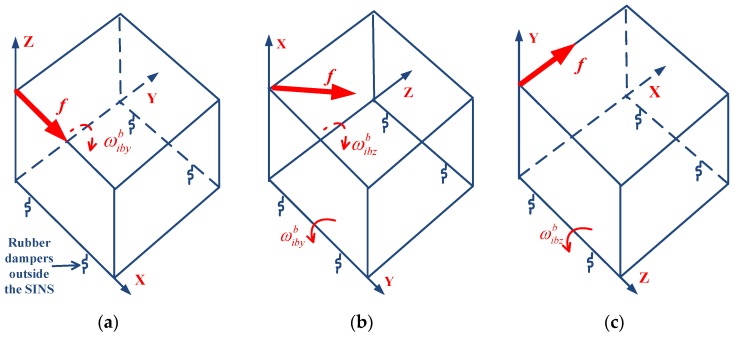

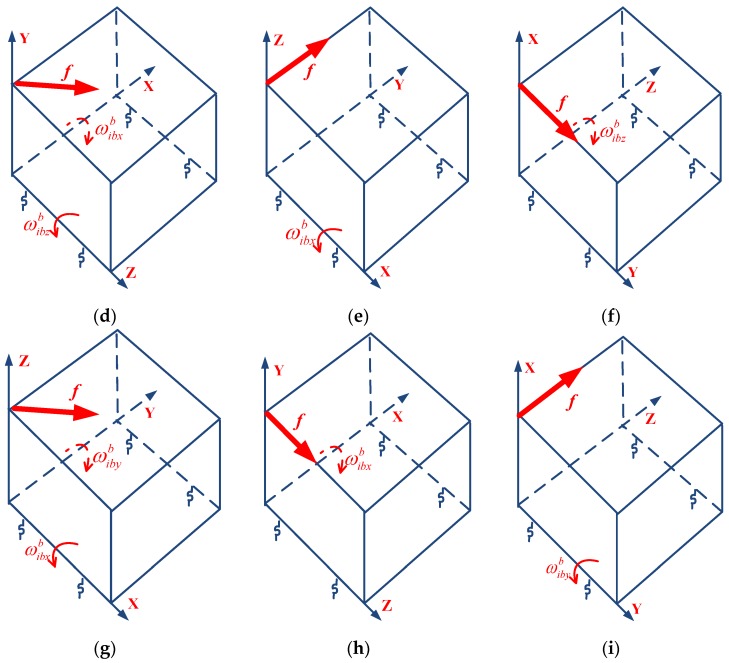
Linear vibration environments generation for the optimal observations of the g-sensitive misalignment parameters: (**a**) $\tau_{xyx}$; (**b**) $\tau_{xyy}$ and $\tau_{xzz}$; (**c**) $\tau_{xzx}$; (**d**) $\tau_{yxx}$ and $\tau_{yzz}$; (**e**) $\tau_{yxy}$; (**f**) $\tau_{yzy}$; (**g**) $\tau_{zxx}$ and $\tau_{zyy}$; (**h**) $\tau_{zxz}$; (**i**) $\tau_{zyz}$.

Intuitively, if the linear vibration directions are respectively along *X*-axes as shown in Figure 3a,c, both the values of Equation (25) will reach their maximums accordingly. Specifically, they are approximately optimal observations for $\tau_{xyx}$ and $\tau_{xzx}$. If the linear vibration direction is along the diagonal of the *YZ* plane as shown in Figure 3b, both the values of Equation (26) will reach their maximums. Namely, this is an approximately optimal observation for $\tau_{xyy}$ and $\tau_{xzz}$.

## 4. Experiment Results

Linear vibration experiments are conducted to calibrate the g-sensitive misalignments of the RLG triad of a high-precision navigation-grade SINS. The SINS is specially designed for the aircraft. The SINS includes three mechanically dithered gyroscopes with a bias stability of 0.005 deg/h (1σ) and three quartz accelerometers with a bias stability of 20 μg (1σ). Before the vibration experiments, we first calibrated the scale factor constants, misalignments, and biases of gyroscopes and accelerometers in the laboratory. The scale factor of the RLG is very stable, with an error of less than 1 ppm, and even as small as 0.1 ppm [21]. Compared with the errors caused by g-sensitive misalignments, the scale factor error of the RLG is so small that it can be ignored. In addition, the size effect parameters and nonlinear coefficients including square coefficients and cross-coupling coefficients of accelerometers should also be calibrated in advance.

**Figure 4:**
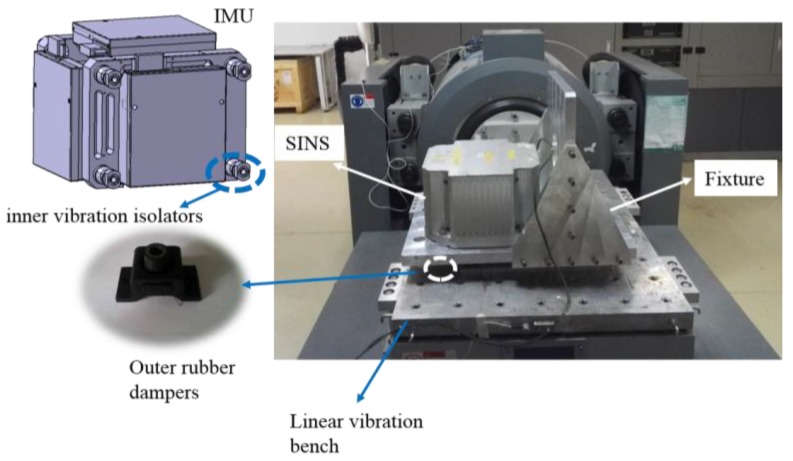
Equipment installation.

As shown in Figure 4, the SINS is directly mounted to a fixture, and the fixture is mounted to the vibration bench through four outer rubber dampers. The rubber dampers are located under the fixture. The fixture is L-shaped, and has two planes including a horizontal plane and a vertical plane. By fixing the SINS to the horizontal and vertical planes of the fixture, we can perform a vibration test on each axis of the SINS. In addition, because the inertial navigation system is a strapdown inertial navigation system, there is no special requirement for SINS installation accuracy on the vibration bench. The vibration bench is a THV710A linear vibration table, which is electro-dynamic. The sine trust and random trust of the vibration bench are 50.0 kN and 50.0 kN (Root Mean Square, RMS), respectively. The frequency range of the vibration bench is from 1 to 2500 Hz. The natural frequency of the selected outer rubber dampers is approximately in the bandwidth of 10~20 Hz. Compared with the SINS’s own inner vibration isolators, the selected outer rubber dampers are far softer, so there will be a large-amplitude angular rate sensed by the RLG triad due to the vibration acceleration acting on the SINS. Vibration experiments are performed on the SINS with approximately optimal observations, as shown in Figure 3. In order to increase the product of specific force and angular rate regarding parameter sensitivity, the amplitude and frequency of vibration are set as 1.5 g and 20 Hz, which is close to the natural frequency of the outer rubber dampers.

According to the above calibration steps, the unknown parameters are solved by Equation (22). The estimates of the component values of τ are listed in Table 1. Taking $\tau_{yxy}$ as an example, whose value is 0.5108 arc-second/g, this means that 1 g specific force along the *Y*-axis causes about 0.5 arc-second of misalignment angle of the *Y* RLG dither axis toward the *X*-axis. Note that coefficients $\tau_{ijj}$ (*i* = *x*, *y*, *z*; *j* = *x*, *y*, *z*; *i* ≠ *j*) account for the lateral anisoelasticity of the *i* RLG dither axis, whose values are close to each other. In addition, the sign of $\tau_{yxx}$ and $\tau_{yzz}$ is positive rather than negative due to the installation relationship of *Y* RLG.

**Figure 5:**
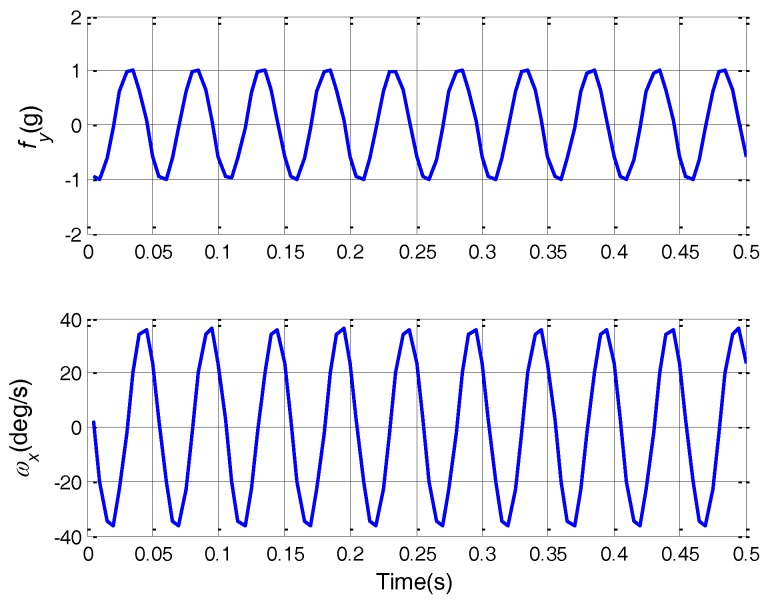
Acceleration and angular rate curves.

To verify the effect of g-sensitive misalignments compensation, a linear vibration test is conducted. The SINS first carries out the alignment process for 15 min. Then the linear vibration along the *Y*-axis in the *XY* plane is applied to the SINS, lasting about 10 min. The SINS works on pure inertial navigation mode during the vibration. Figure 5 shows the details of $f_{y}^{b}$ and $\omega_{ibx}^{b}$ within 0.5 s. The curves in this figure are plotted according to the IMU gyro output and accelerometer output. By inspection of the figure, we can obtain the frequency, amplitude, and phase information of $f_{y}^{b}$ and $\omega_{ibx}^{b}$. The frequency of $f_{y}^{b}$ and $\omega_{ibx}^{b}$ is 20 Hz. The amplitudes of $f_{y}^{b}$ and $\omega_{ibx}^{b}$ are about 1 g and 36 deg/s. Due to the damping effect of the outer rubber dampers, there is a phase difference between $f_{y}^{b}$ and $\omega_{ibx}^{b}$, whose value is about 3π/5. Hence, the representations of $f_{y}^{b}$ and $\omega_{ibx}^{b}$ are given by:

(27) $$f_{y}^{b}\approx g\cdot \sin\left(40\pi t\right),\omega_{ibx}^{b}\approx \frac{\pi }{5}\sin\left(40\pi t-\frac{3\pi }{5}\right)$$

In this condition, the equivalent gyro drift regarding $f_{y}^{b}$ and $\omega_{ibx}^{b}$ is given by:

(28) $$\delta \omega \approx \tau_{yxy}f_{y}^{b}\omega_{ibx}^{b}=\tau_{yxy}\frac{1}{2}\times g\times \frac{\pi }{5}\times \left[\cos\frac{3\pi }{5}-\cos(80\pi t+\frac{3\pi }{5})\right]$$

**Figure 6:**
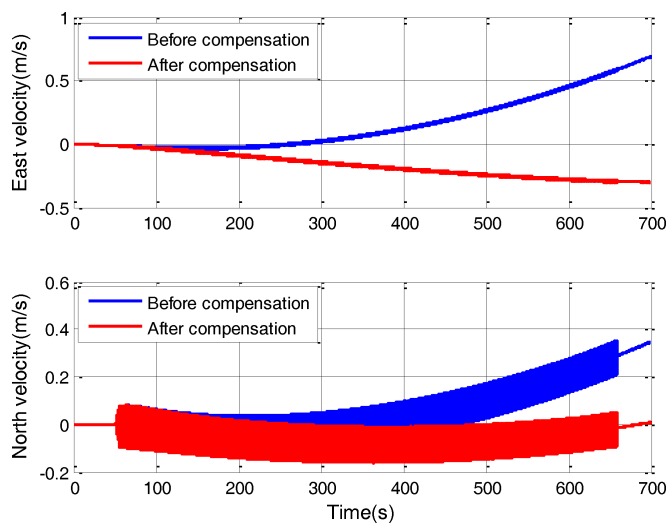
Velocity error curves.

The direct component of Equation (28) is the constant equivalent gyro drift. The value is about 0.05 deg/h, which is a great value for high-accuracy SINS. The equivalent gyro drift will further introduce great velocity error, as shown in Figure 6. After g-sensitive misalignments compensation, the velocity error significantly decreases. The percentage of velocity error decrease is about 61%.

**Figure 7:**
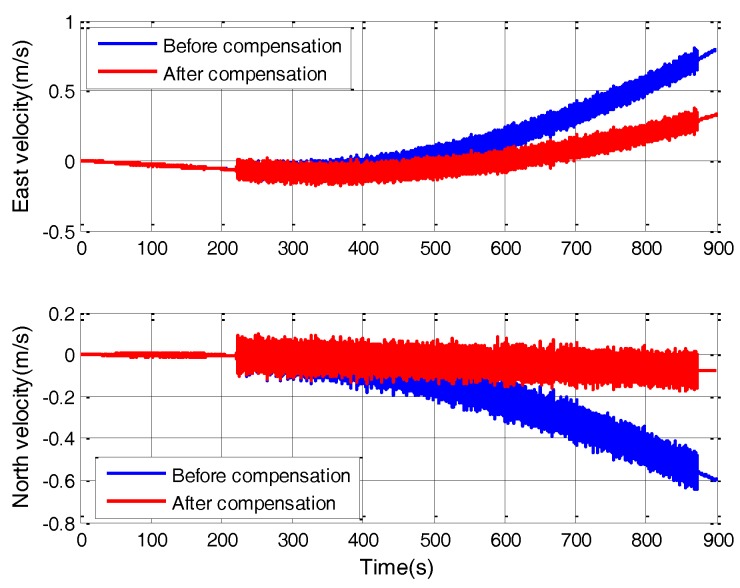
Velocity error in random vibration.

In order to further verify the effect of g-sensitive misalignments compensation, other 27 sets of vibration experiments including fixed frequency vibration and random vibration are performed on the SINS. The amplitude and frequency of fixed frequency vibration are listed in Table 2. For random vibration, 0.7 g root mean square (RMS) vibration acceleration is set with constant power spectral density (PSD) in a frequency range from 10 to 60 Hz. The vibration direction is along the diagonal of the SINS, which makes the acceleration act on the SINS’ multiple axes at the same time. The SINS first carries out the alignment process for 15 min before the vibration test. Each vibration experiment lasts about 10 min. For the high-accuracy SINS, compared with the error caused by g-sensitive misalignments in vibration, the influence of other error sources, e.g., gyro bias and accelerometer bias, can be ignored within 10 min, which is much less than the Schuler period, i.e., 84.4 min. So, the major errors during the vibration for 10 min are the g-sensitive misalignments. The choice of vibration time is for error analysis. The SINS works on pure inertial navigation mode during the vibration. The evaluation index is the percentage of velocity error decrease after g-sensitive misalignments compensation. Figure 7 depicts the velocity error curves in one of the random vibration tests. After g-sensitive misalignments compensation, the velocity error decreases by 66.1%.

**Figure 8:**
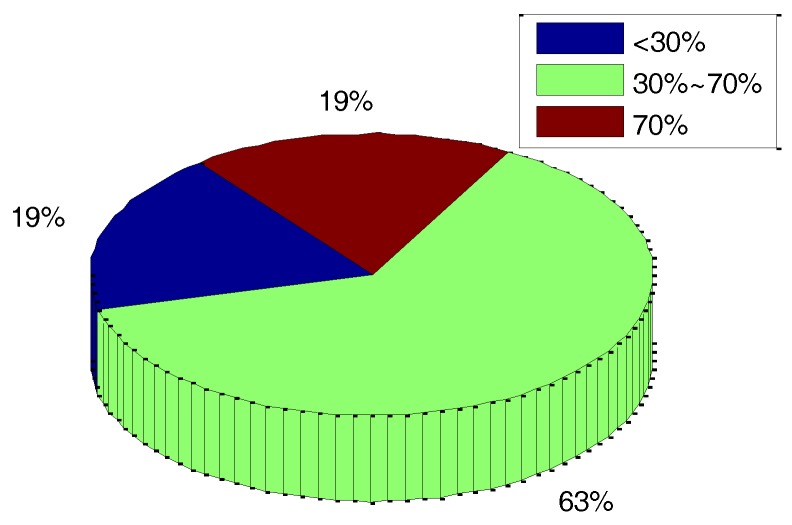
Distribution of the percentage of velocity error’s decrease.

All of the percentages of velocity error decrease corresponding with the 27 sets of vibration experiments are listed in Table 2. A corresponding statistic pie chart is shown in Figure 8. The statistical results show that the proportion of velocity error decreasing by more than 30% in the total results reach about 82% after g-sensitive misalignments compensation. As shown in Table 2 and Figure 8, though the proportion of velocity error decreasing by less than 30% is about 18%, which is affected by random measurement error to a certain extent, the systematic errors caused by g-sensitive misalignments can be compensated for to a large extent. 

## 5. Conclusions

In this paper, a novel method to calibrate g-sensitive misalignments of an RLG triad in linear vibration environments is proposed to improve the SINS accuracy for applications in spacecraft and other aerospace systems. Compared with the traditional calibration method in a gravitational field, the method presented in this paper is based on linear vibration experiments to simulate the harsh environment during aircraft flight. The derived mathematical model of g-sensitive misalignments can be used to relate attitude error to specific force and angular rate. With approximately optimal observations, g-sensitive misalignments are obtained by least squares estimations. Vibration experiments show the validity of the proposed method.

## Figures and Tables

**Table 1:** Parameter estimates.

Parameter	Value (Arc-Second/g)	Parameter	Value (Arc-Second/g)	Parameter	Value (Arc-Second/g)
$\tau_{xyx}$	0.329	$\tau_{yxx}$	0.883	$\tau_{zxx}$	−1.353
$\tau_{xyy}$	−1.679	$\tau_{yxy}$	0.511	$\tau_{zxz}$	−0.292
$\tau_{xzx}$	−0.418	$\tau_{yzy}$	−0.401	$\tau_{zyy}$	−1.052
$\tau_{xzz}$	−1.684	$\tau_{yzz}$	1.100	$\tau_{zyz}$	0.237

**Table 2:** Velocity error decreases.

Amplitude and Frequency	Percentage of Velocity Error’s Decrease		
1 g, 10 Hz	49.5%	61.2%	22.4%
1.5 g, 10 Hz	45. 9%	41.4%	45.3%
1.5 g, 10 Hz	20.8%	77.7%	76.0%
0.5 g, 20 Hz	67.6%	88.2%	45.5%
0.5 g, 20 Hz	54.2%	52.9%	21.0%
1 g, 20 Hz	33.7%	41.9%	52.8%
1 g, 20 Hz	48.8%	31.7%	46.8%
2 g, 20 Hz	82.2%	88.7%	21.3%
Random	62.1%	29.4%	66.1%
